# Spermicidal and Contraceptive Potential of Desgalactotigonin: A Prospective Alternative of Nonoxynol-9

**DOI:** 10.1371/journal.pone.0107164

**Published:** 2014-09-22

**Authors:** Debanjana Chakraborty, Arindam Maity, Tarun Jha, Nirup Bikash Mondal

**Affiliations:** 1 Department of Chemistry, Indian Institute of Chemical Biology, Council of Scientific and Industrial Research, Jadavpur, Kolkata, West Bengal, India; 2 Department of Pharmaceutical Technology, Division of Medicinal and Pharmaceutical Chemistry, Jadavpur University, Kolkata, West Bengal, India; University of Nevada School of Medicine, United States of America

## Abstract

Crude decoction of *Chenopodium album* seed showed spermicidal effect at MIC 2 mg/ml in earlier studies. Systematic isolation, characterization and evaluation revealed that the major metabolite Desgalactotigonin (DGT) is the most effective principle in both *in vitro* and *in vivo* studies. The *in vitro* studies comprises (a) rat and human sperm motility and immobilizing activity by Sander-Cramer assay; (b) sperm membrane integrity was observed by HOS test and electron microscopy; (c) microbial potential was examined in *Lactobacillus* broth culture, and (d) the hemolytic index was determined by using rat RBCs. The *in vivo* contraceptive efficacy was evaluated by intra uterine application of DGT in rat. Lipid peroxidation and induction of apoptosis by DGT on human spermatozoa were also studied. The minimum effective concentration (MEC) of DGT that induced instantaneous immobilization *in vitro* was 24.18 µM for rat and 58.03 µM for human spermatozoa. Microbial study indicated DGT to be friendly to *Lactobacillus acidophilus*. Implantation was prevented in DGT treated uterine horn while no hindrance occurred in the untreated contra lateral side. At the level of EC_50_, DGT induced apoptosis in human spermatozoa as determined by increased labeling with Annexin-V and decreased polarization of sperm mitochondria. Desgalactotigonin emerged 80 and 2×10^4^ times more potent than the decoction and Nonoxynol-9 respectively. It possesses mechanism based detrimental action on both human and rat spermatozoa and spares lactobacilli and HeLa cells at MEC which proves its potential as a superior ingredient for the formulation of a contraceptive safer/compatible to vaginal microflora.

## Introduction

The world population, which was 2.5 billion in 1950, has crossed the 7 billion mark [1] in 2012 and is projected to touch 9.4 billion by 2050 [2]. Anticipating that 86% of the global population will live in less developed countries by the year 2050 [3], the present-day scenario of world population looks alarming. The demographers and social scientists have recommended drastic family planning programs and emphasized that contraception is the only means to combat this devastating problem; they therefore felt, among others, an urgent need for developing options that allow women to prevent or delay their pregnancies [4]. Although a number of contraceptive methods have been developed, the acceptability of these methods has quite often been limited by their associated untoward side effects, failure rate or irreversibility [5]. Spermicides are biologically obvious ways of interrupting fertility and have the advantage that they do not require highly skilled personnel for their prescription and use. Thus research has focused on the development of safe, highly effective and inexpensive spermicidal agents as one of the several alternative methods for family planning [6].

The currently available spermicidal contraceptive formulations, mostly based on the mixture of oligomers nonoxynol-9 or N-9, [7], [8] are effective but their repeated use is associated with vaginal/cervical irritation or even ulceration [9] and disturbance of the normal vaginal microflora; this facilitates microbial infection and renders the subject susceptible to sexually transmitted diseases (STDs) [10]. Several European nations have banned or restricted the use of N-9 related compounds on the basis of health risks and potential environmental toxicity [11], [12]. The limitations of N-9 to protect sexually transmitted diseases [13] have encouraged researchers to develop better alternatives that would have dual function of contraception and STD protection for women.

Our group has made concerted efforts to develop novel spermicidal cum microbicidal molecules from synthetic [14], [15] and natural [16], [17] sources. Previous studies on *Chenopodium album* revealed that the aqueous decoction of the seeds has *in vitro* sperm immobilizing activity [18], [19], [20]. As the healthcare industry moves towards using either pure compounds or mixtures whose individual components meet safety standards, we undertook the program of separating the active constituent(s) of the decoction *of C. album* seeds. We have thereby succeeded in isolating the active components. In this article we wish to report both the spermicidal and microbial potential of the isolated molecule(s) which appears to possess the dual function of contraception and STD protection for women.

## Materials and Methods

### Plant material

Matured fruits of *Chenopodium album* (Linn) were collected from the medicinal plant garden of R. K. Mission, Narendrapur, Kolkata, during April 2012. As the plant is not an endangered or protected species rather a small herb that grows all over the garden as a common agricultural weed thus no specific permissions were required for its collection. This plant material was authenticated by Dr. Debjani Basu, Asst. Director, Botanical Survey of India, Howrah (West Bengal, India). A voucher specimen (No. 786) was deposited in the Steroid and Terpenoid Chemistry Department, Indian Institute of Chemical Biology. The black seeds were segregated from the pericarp of the fruits and then ground in an industrial blender.

### Preparation of extract

Powdered seeds (3 Kg) were subjected to successive percolation with (i) petroleum ether (60–80°C), (ii) chloroform, (iii) methanol, and (iv) methanol: water (1∶1). The solvents were distilled off under reduced pressure (temperature <50°C) using a rotary evaporator (Eyela, Tokyo, Japan). Removal of inorganic salts from the methanol extract was effected by partitioning between n-butanol and water. The n-butanol part was concentrated by reduced pressure distillation to yield 35 g of greenish brown mass.

### Purification of extract

The greenish brown mass obtained from the n-butanolic portion was passed through silica gel column (60–120 mesh size) and eluted with solvents in an increasing order of polarity starting with chloroform and ending with methanol. The fractions obtained by column chromatography from different ratios of chloroform: methanol was subjected to screening for spermicidal activity. The active fractions were further purified through repeated column chromatography in conjunction with thin layer chromatography. This ultimately yielded four products, the major one with eluent CHCl_3_: MeOH (75∶25) and the minor three were with (70∶30), (65∶35) and (50∶50) respectively. The major product which was crystallized from MeOH and characterized as Desgalactotigonin (yield 0.004%, on dry weight basis of the plant material) via spectroscopic analysis, viz. mass, ^13^C NMR, and ^1^H NMR spectroscopy followed by comparison with data reported in the literature [21]. It is worthy of mention that this compound is being isolated for the first time from *Chenopodium album*. The minor products were also characterized by spectral analysis as glucuronopyranosyl analogues of oleanolic acid viz. (i) 3-*O*-β-D-Glucuronopyranosyl oleanolic acid, (yield 0.0001%) [22] (ii) 3-*O*-[β-D-Glucuronopyranosyl]-28-*O*-β-D-g-lucuronopyranosyl oleanolic acid (yield 0.0006%) and (iii) 3-*O-*[3′-*O*-(2″-*O*-Glycolyl)-glyoxylyl β-D-glucuronopyranosyl] oleanolic acid (yield 0.0005%) [23]. These glucuronopyranosyl analogues of oleanolic acid were also present in trace amounts in methanol: water (1∶1) fraction. However, these were not taken into account as they showed less activity in comparison to desgalactotigonin. (See Data S1).

**Figure 1:**
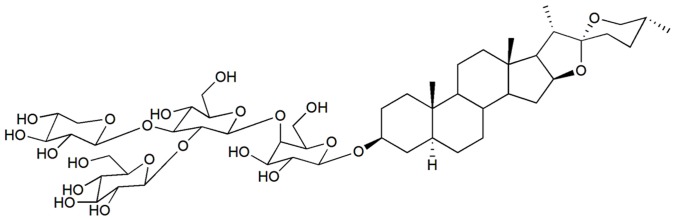
Structure of Isolated Compound. Structure of desgalactotigonin (DGT).

Desgalactotigonin (Fig.1) is an amorphous solid, mp (235-237°C); Q-TOF Mass (m/z) 1057 [M + Na]^+^; ^1^H NMR (Pyridine-*d*_5,_ 600 MHz) δ 0.51 (1H, m), 0.64 (3H, s), 0.70 (3H, d, *J* = 4.8 Hz), 0.82 (4H, s), 0.89 (1H, m), 1.05 (3H, m), 1.15 (5H, d, *J* = 7.2 Hz), 1.38 (4H, m), 1.61 (8H, m), 1.79 (2H, m), 1.99 (2H, m), 3.61 (2H, m), 3.92 (5H, m), 4.08 (6H, m), 4.19 (1H, m), 4.24 (3H, m), 4.43 (3H, m), 4.58 (3H, m), 4.89 (1H, d, *J* = 7.2 Hz), 5.20 (1H, d, *J* = 8.4 Hz), 5.25 (1H, d, *J* = 7.8 Hz), 5.43 (1H, s), 5.59 (1H, d, *J = *7.2 Hz); ^13^ C NMR [Pyridine-*d*_5_, 150 MHz] δ 12.5 (CH_3_), 15.3 (CH_3_), 16.8 (CH_3_), 17.7 (CH_3_), 21.5 (CH_2_), 29.1 (CH_2_), 29.5 (CH_2_), 30.1(CH_2_), 30.8 (CH), 32.0 (CH_2_), 32.4 (CH_2_), 32.6 (CH_2_), 35.0 (CH_2_), 35.5 (CH), 36.0 (C), 37.4 (CH_2_), 40.4 (CH_2_), 41.0 (C), 42.2 (CH), 44.9 (CH), 54.6 (CH), 56.7 (CH), 60.5 (CH_2_), 62.7 (CH_2_), 63.2 (CH), 63.2 (CH_2_), 67.1 (CH_2_), 67.6 (CH_2_), 70.7 (CH), 70.9 (CH), 71.2 (CH), 73.4 (CH), 75.3 (CH), 75.6 (CH), 75.8 (CH), 76.5 (CH), 77.6 (CH), 77.9 (CH), 78.0 (CH), 78.9 (CH), 79.0 (CH), 80.2 (CH), 81.4 (CH), 81.6 (CH), 86.9 (CH), 102.6 (CH), 105.1 (CH), 105.2 (CH), 105.4 (CH), 109.5 (C).

### Chemicals

Chemicals and solvents were of analytical grade and mostly procured from Merck India Ltd. LIVE/DEAD sperm viability kit was from Life Technologies (Invitrogen, India Pvt Ltd) and the other chemicals were from Sigma (St Louise, MO, USA). JC-1 and FITC-Annexin-V/PI assay kits were purchased from Life Technologies (Molecular Probes, USA) and Calbiochem (USA) respectively. Thiobarbituric acid (TBA) and trichloroacetic acid (TCA) were purchased from Merck Specialities Pvt Ltd (Mumbai, India).

### Animals

Adult Sprague-Dawley rats (230–270 g) were collected from the animal house of the Indian Institute of Chemical Biology. The animals were maintained under standard husbandry conditions (12∶12, dark/light cycle) and fed with standard pellet diet; water was supplied ad libitum. All experiments were performed in accordance with the guidelines recommended and approved by the Indian Institute of Chemical Biology's Animal Care and Use Committee of Laboratory Animals (Animal Ethics Committee) registered with the Ministry of Social Justice &Empowerment for breeding, maintenance and experimentation of animals (IICB Reg. No. is 147/1999/CPCSEA, renewed in 2002 and 2005).

### Isolation of rat caudal sperm

Males of proven fertility were used for sperm collection following sexual abstinence. The animals were necropsied following anesthesia with ketamine (100 mg/kg body weight). The caudal portion of the epididymis was dissociated and minced in 2.5 ml of BWW medium (94 mM NaCl, 4.7 mM KCl, 1.7 mM CaCl_2_, 1.2 mM KH_2_PO_4_, 1.2 mM MgSO_4_·7H_2_O, 25 mM NaHCO_3_, 0.5 mM sodium pyruvate, 19 mM sodium lactate, 5 mM glucose, 0.4% BSA, 0.1% antibiotic (penicillin/streptomycin) solution, pH 7.2) [24]. The collected spermatozoa exudates were then suspended in the same medium. The spermatozoa were incubated for 30 min under oil cover in a CO_2_ chamber. Sperm count was done using a Makler's chamber. Highly motile sperm suspensions with a sperm count of 20–25×10^6^/ml were used for further work.

### Human spermatozoa

Fresh human semen samples, obtained by masturbation into a sterile vial from healthy, young fertile donors, were liquefied for 45 min at 37°C and used for in vitro spermicidal assays. Samples having >65×10^6^/ml sperm count with >70% motility and normal sperm morphology were used in the study. The study protocol was approved by the Institutional Ethics Committee of Institute of Reproductive Medicine, Kolkata, governed by Indian Council of Medical Research, New Delhi (Ref. No.IRM/HEC/BNC/25-01-2013). Written informed consent was obtained from all the participants prior to enrolling in the study. The highly motile spermatozoa with forward motility were washed with BWW medium and separated from immotile or sluggishly motile spermatozoa by ‘swim up’ technique and semen characteristic, pH, motility, morphology were determined on the World Health Organization guidelines [25] and finally resuspended in pre-equilibrated BWW medium to obtain working spermatozoa suspension having concentration of 30–35×10^6^ cells/ml.

### Assessment of spermicidal activity

The spermicidal activity of desgalactotigonin (DGT) was assayed according to the modified Sander–Cramer method [26], [27]. Varying concentrations of DGT solution (0, 5, 10, 15, 20, 25, 30 µM in case of rat and 0, 15, 30, 45, 60, 65 µM for human) were prepared in physiological saline and 100 µl of each was mixed with a 20 µl aliquot of sperm suspension (25×10^6^/ml). The specimens were examined under a phase contrast microscope (×100) after 20 s of treatment and counted for motile spermatozoa. The sperm that lost complete motility within 20 s were subsequently tested for motility revival. About 250 µl of Baker's buffer (glucose 3%, Na_2_HPO_4_·2H_2_O 0.31%, NaCl 0.2%, KH_2_PO_4_ 0.01%) was used to show total immobilization of the spermatozoa by incubating at 37°C for 60 min and observing for regeneration of any kind of motility. The minimum concentration of DGT that caused 100% immobilization of spermatozoa within 20 s with no revival of motility after subsequent 60 min of incubation in Baker's buffer at 37°C was considered to be the minimum effective concentration (MEC).

### Hypo-osmotic swelling test

Control and DGT-treated (at MEC, i.e. 24.18 µM for rat and 58.03 µM for human) spermatozoa suspensions were exposed to HOS solution (75 mM fructose, 20 mM sodium citrate) for at least 30 min at 37°C. After thorough mixing, a drop of mixture was placed on a glass slide and covered with a cover slip to detect changes in sperm membrane integrity [28], [29], [30], [31]. The number of spermatozoa showing characteristic tail curling or swelling was counted under a phase contrast microscope (×400).

### Sperm viability test by fluorescent staining

Sperm viability was assessed by using a sperm viability kit according to the manufacturer's instructions, where live spermatozoa fluoresced green (SYBR-14 dye) and dead spermatozoa fluoresced red [propidium iodide (PI)]. DGT treated (for rat at MEC 24.18 µM and human at MEC 58.03 µM) and untreated sperm samples were subjected to sperm viability assessment [32]. Sperm count was taken from 200 spermatozoa under a phase contrast microscope (×200).

### Assessment of irritation potential through hemolytic studies

The irritation potential of the compound was estimated using an RBC hemolytic assay. The RBC from rat blood was isolated by repeated washing (three times) with isotonic phosphate buffered saline (PBS; pH 7.4). These were mixed with varying concentrations of DGT, 0.1% of Triton X-100 and PBS, and used as treated, positive control and negative control, respectively. The test samples were prepared in PBS and incubated at 37°C for 30 min with occasional mild shaking to achieve complete hemolysis. The reaction was quenched in ice, the samples were centrifuged at 1500 rpm for 2 min, and the supernatants were used for spectrophotometer reading at 576 nm. The percentage hemolysis was then calculated by the following formula [33]: %H = 100% (Abs – Abs_control_)/(Abs_100_ − Abs_control_), where, Abs is the absorbance of the sample, Abs_control_ is the absorbance of the control sample (negative control) and Abs_100_ is the absorbance of the sample where 100% hemolysis occurred. Hemolytic index (50% hemolysis) was determined by comparing control and positive control.

### Evaluation of effect on *Lactobacillus acidophilus* NCIM 2285

#### Inoculum preparation

Bacterial colonies were grown in MRS broth at 37°C for 36 h, adjusted to 0.5 McFarland standard, [34] and diluted with 0.9% sterile saline to a final count of 1.5×10^6^ CFU/ml.

The compound (DGT) was first dissolved in 0.4% DMSO, diluted with MRS broth media to give a final concentration of 1000 µg/ml, and then serially diluted two fold to obtain different concentration ranges [35]. An aliquot (0.1 ml) of standardized suspension of bacteria (1.5×10^6^ CFU/ml) was added to each well (96 microplate) containing the test compound at a final concentration of 0–1000 µg/ml, and incubated at 37°C for 36 h [36]. Beside each of test sample, one negative control well was taken which contained liquid broth culture without any test sample. N-9 (Sigma-Aldrich) was used as positive control. The absorbance of the solution was measured at 540 nm.

### Contraceptive efficacy

The contraceptive efficacy of the compound was assayed in rats via intrauterine administration of DGT and subsequent evaluation of mating outcomes. Sexually mature cyclic adult Sprague-Dawley rats (n = 10) were subjected to light ether anesthesia on the day of proestrous phase of their cycle, then one very small mid-ventral abdominal incision was made through which the uterine horns were gently pulled out carefully. In one horn, 50 µl of DGT solution (3000 µg/ml of sterile physiological saline) was introduced through a tuberculin syringe fitted with a 24-G needle that penetrated through the cervical end (treated horn). The contralateral uterine horn received 50 µl of sterile physiological saline (control horn). The incision was closed by suture and Mitchel's clip. The animals were maintained in individual cages and divided into two groups with five animals per group. In the evening, they were exposed to males of proven fertility in 1∶1 ratio. In the next morning the presence of sperm in vaginal lavage confirmed mating. Mated rats of one group were sacrificed on Day 10 of gestation. The uterine horns were examined and the number of implantation sites/fetus was counted. Animals of the other groups were allowed to deliver pups. After parturation, pups were examined visually for any abnormalities.

### Ultrastructural study

#### Transmission electron microscopy

Control and DGT treated human sperm suspensions were fixed in 2.5% glutaraldehyde in 0.1% cacodylate buffer for 3 days following the procedure of Niksirat et al. [37]. After three successive washings in buffer, overnight post-fixation was done using 4% osmium tetroxide solution. Three further washings in buffer were done and samples were dehydrated through graded series of acetone. Finally the samples were embedded in spur resins and polymerized for 48 h. Ultra thin sections were cut, stained with uranyl acetate and lead citrate, and observed under transmission electron microscope (JEOL, Tokyo, Japan) operating at 80 kV. Fifty (50) spermatozoa were scanned from treated and untreated groups for the intactness of sperm head membranes; photograph of one representative sperm from each group has been presented.

#### Scanning electron microscopy

For scanning electron microscopic (SEM) observation, human spermatozoa were fixed with 2.5 % glutaraldehyde in 0.1% cacodylate buffer. Next, samples were rinsed three times in filtered f/2 medium and immobilized by spreading onto a poly-L-lysine coated glass slide. Samples were then post fixed by 2% osmium tetroxide and dehydrated in graded series of acetone, with 15 min incubation at each step. After drying by the critical point method (CPD2, Pelco TM), glass slides were mounted on an aluminum stub using carbon conductive tape and a silver paste. Specimens were gold coated (SEM Coating Unit E 5100, Polaron) and observed in a field emission scanning electron microscope (JEOL JSM 7401-F).

### Determination of lipid peroxidation on human sperm

Lipid peroxidation was measured to determine the extent of oxidative damage induced by DGT on the human sperm membrane as indicated by malondialdehyde (MDA) adduct formation. DGT was serially diluted with BWW medium to make solutions of final concentrations ranging between 29.01 µM and 106.38 µM and treated with the sperm suspension at 5∶1 ratio. The sperm suspension (1 ml of 25–30×10^6^ cells/ml solution), treated with or without different concentration of DGT, was mixed with 2 ml of TBA-TCA reagent (15% w/v TCA, 0.375% w/v TBA, and 0.25 M HCl). The mixture was boiled in water bath for 30 min. After cooling, the suspension was centrifuged at 1500 g for 15 min. The supernatant was separated and the absorbance was measured at 535 nm. The result was expressed as simple concentration of MDA (nmol/10^8^ sperm) as determined by specific absorbance coefficient (1.56×10^5^/mol per cm^3^) [38], [39]. MDA produced (µmol/ml) = [OD×10^6^×total volume (3 ml)]/[1.56×10^5^×test volume (1 ml)].

### Cytotoxicity study against the human cervical cell line (HeLa) by MTT assay

Colorimetric assay can be done using the 3-(4,5-dimethylthiazol-2-yl)-2,5-diphenyltetrazolium bromide (MTT) assay. It was used for evaluation of cytotoxicity of spermicidal compound against the HeLa cell line. In each well of a 96 well culture plate 2×10^5^ cells were seeded in triplicate. The cells were incubated for 24 h in a 5% CO_2_ incubator at 37°C and then exposed to various concentrations of DGT. At the end of the treatment the drug containing medium was removed and 20 µl of 5 mg/ml MTT dissolved in the same medium was added to each well. The plate was further incubated for an additional 5 h. After removing the supernatant at the end of incubation, the resultant intracellular formazan crystals were solubilised with DMSO and the absorbance of the solution was measured at 595 nm using an ELISA reader. Absorbance (O.D) of the medium containing the different concentrations of the compound and MTT reagents was subtracted from the respective experimental sets and then viability was calculated.

### Apoptotic changes in plasma membrane of human spermatozoa

Dual fluorescent labelling with fluorescein isothiocyanate (FITC)-Annexin V and propidium iodide (Calbiochem, USA) was used to study the expression of phosphatidylserine on sperm cell surfaces (apoptotic cells) and the membrane permeabilization of the cells (necrotic cells) respectively. Aliquots of highly motile human sperm (10^7^) in triplicate were incubated in BWW-0.4% bovine serum albumin (BSA) containing 94 mM NaCl, 4.7 mM KCl, 1.7 mM CaCl_2_, 1.2 mM KH_2_PO_4_, 1.2 mM MgSO_4_·7H_2_O, 25 mM NaHCO_3_, 0.5 mM sodium pyruvate, 19 mM sodium lactate, 5 mM glucose, 0.1% antibiotic (penicillin/streptomycin) solution, pH 7.2 at 37°C for 3 h with EC_50_ of DGT. Control tubes contained only BWW medium with 0.4% BSA. After incubation, sperms were washed in 1% BSA in Tyrode's buffer and labeled with fluorescent probes. An apoptosis detection kit (Calbiochem, USA) was used to detect apoptosis in sperm cells by following manufacturer's instruction. The percentages of cells positive for Annexin-V and PI were determined in a flow cytometer (Model BD LSRFortessa, BD Biosciences, USA). Four populations of cells (unstained, FITC stained, FITC + PI stained, and PI stained) were identified [40] and designated as viable, apoptotic, necrotic and dead respectively.

### Apoptotic changes in mitochondria on human sperm cell

The loss of mitochondrial trans-membrane potential (ΔΨm, an early marker for cell apoptosis) was quantified by flow cytometry using the lipophilic cationic dye 5,5′,6,6′-tetrachloro-1,1′,3,3′-tetraethylbenzimidazolylcarbocyanine iodide (JC-1). Concurrently, highly motile human spermatozoa (10^7^ in triplicate) were incubated in BWW-0.4% BSA at 37°C for 3 h with EC_50_ of DGT. Positive control tubes contained 50 µM CCCP and control tubes contained BWW-0.4% BSA. After incubations, 10 µg/ml JC-1 (Molecular probes) was added to sperm from a stock solution of 1 mg/ml in DMSO. Spermatozoa were incubated for another 10 min, washed and re-suspended in Tyrode's salt solution. The cells were finally analysed in a flow cytometer (Model BD LSR Fortessa, BD Biosciences, USA) using an argon laser (488 nm) for excitation, and emissions at 530 nm (green) and 575 nm (red/orange) were quantified using the threshold signal for intact cells.

## Results

### Spermicidal activity

**Figure 2:**
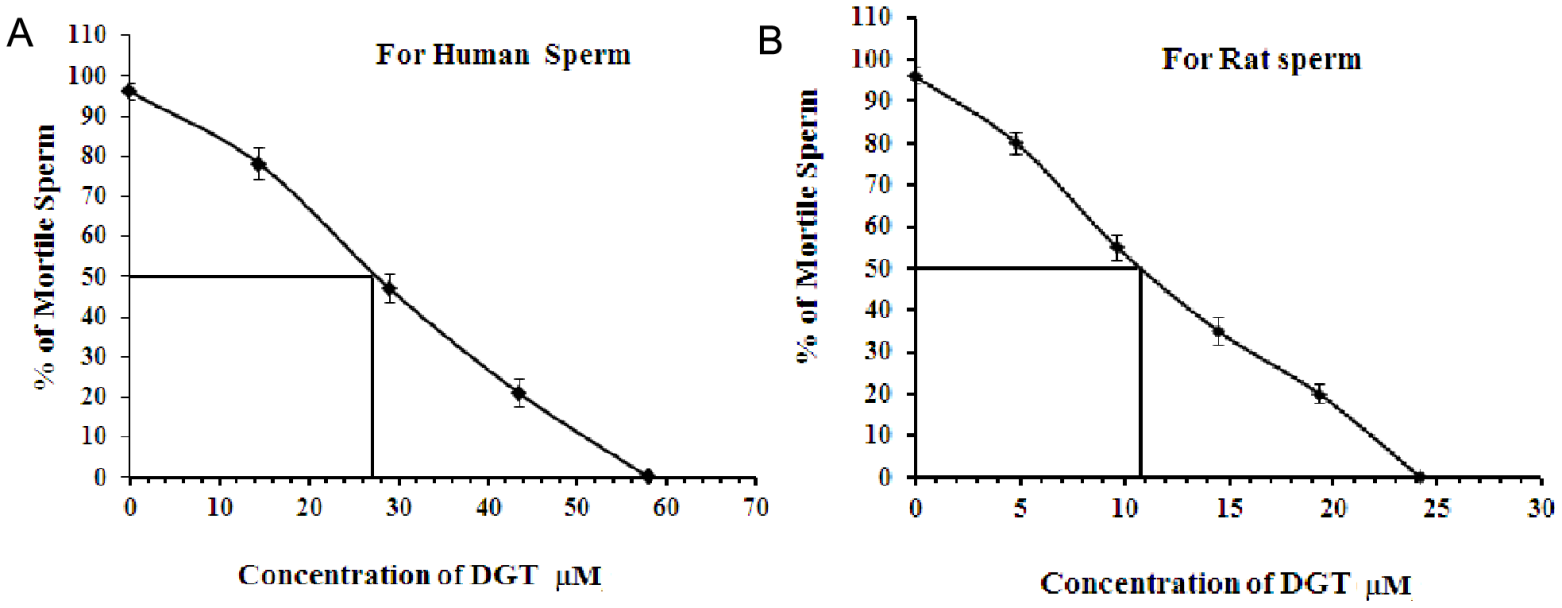
MEC Dose of the compound. Dose-dependent sperm immobilizing activity of DGT. The percentage of motile (A) human spermatozoa and (B) rat spermatozoa were determined after 20s following exposure to the test compounds at different concentrations. All data were adjusted to a normal control motility of 95%. Each point of DGT represents the mean ± SEM. values of five independent experiments.

There was a dose-dependent increase in sperm immobilization with an increase in concentration of DGT. (Fig. 2 A and B) depict the effect of varying concentrations of DGT on both human and rat sperm motility. The MEC that induced 100% immobilization of the spermatozoa in 20 s was 24.18 µM for rat and 58.03 µM for human. At MEC of DGT, no revival of motility was observed after subsequent 60 min of incubation in Baker's buffer at 37°C.

### Sperm membrane integrity

**Figure 3:**
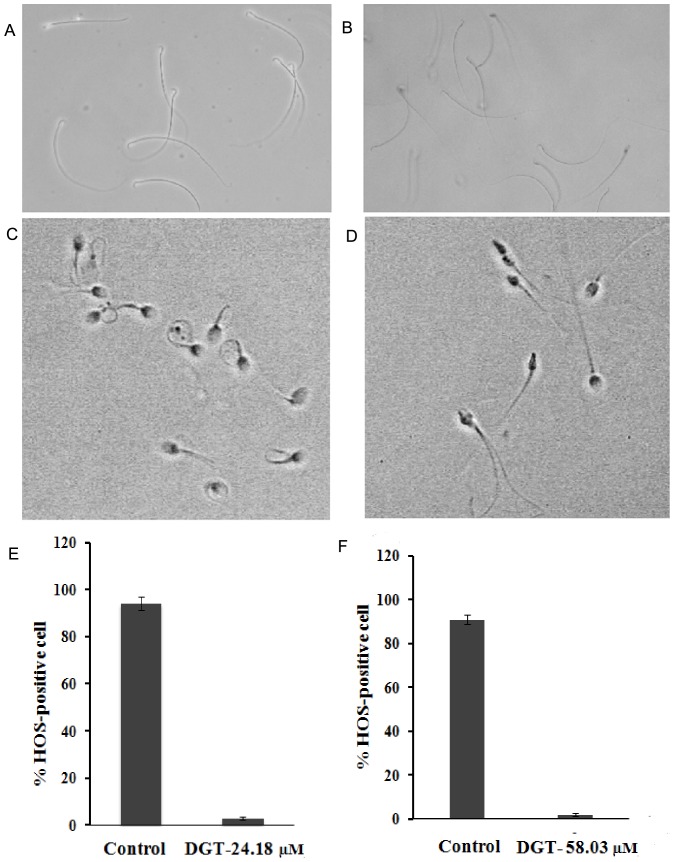
The HOS responsiveness of the sperm, as indicated by tail coiling. HOS responsiveness of rat sperm as tail coiling: (A) Untreated control. (B) DGT-treated rat sperm (24.18 µM) examined under a phase contrast microscope (40×). Response of control (C) and DGT-treated (D) human sperm (58.03 µM) population following exposure to hypo-osmotic solution and evaluation under a phase contrast microscope. Over 90% of control-untreated sperm exhibited HOS response typically characterized by tail coiling, where as sperm exposed to DGT at MEC examined no response under a phase contrast microscope (40×). (E): For rat sperm, (F): For human sperm. Each bar represents the mean ± SEM of five observations (P<0.05).

More than 90% of the control spermatozoa (both rat and human) were HOS responsive, whereas DGT-treated spermatozoa at MEC (both rat and human) was non-HOS responsive as depicted via tail curling (Fig. 3 A, B and E for rat and Fig. 3 C, D and F for human). Loss of HOS responsiveness indicated compromised sperm membrane integrity post DGT treatment, anticipating overall loss of sperm-membrane physiology.

### Sperm viability test by fluorescent staining

**Figure 4:**
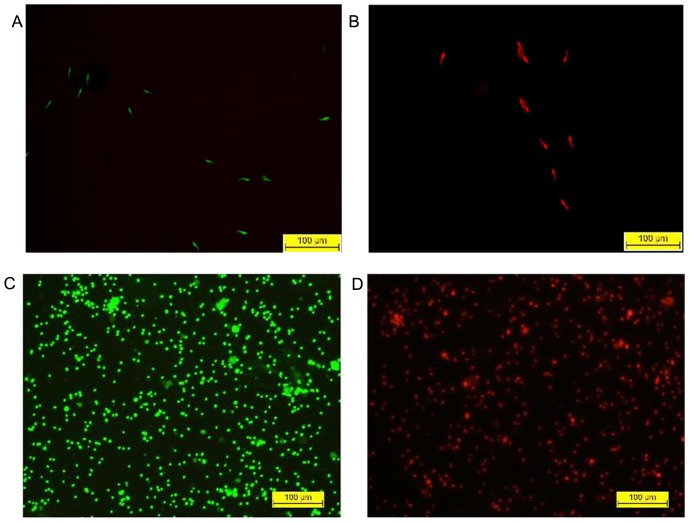
The sperm viability, as assessed by a fluorescent staining method. Rat Sperm viability assessment by SYBR-14/PI staining. (A) Control rat sperm appear green due to uptake of SYBR14 only; (B) DGT-treated rat sperm appear red due to uptake of PI when observed under a fluorescence microscope. Overlaid fluorescence images of (C) control and (D) DGT-treated human spermatozoa, dual stained with SYBR-14 and propidium iodide to distinguish green-fluorescing live from red-fluorescing dead spermatozoa.

The fluorescent green (Sybr-14) and red (PI) dyes differently stain live and dead sperm, respectively. At MEC in case of rat (24.18 µM), treated sperm were all dead, which fluoresced red, and control sperm fluoresced green (Fig. 4A–B). In the control set (without DGT) of human spermatozoa, 92% fluoresced green and were viable, while cells treated at MEC (58.03 µM), which fluoresced red (Fig. 4C–D), were all dead.

### Irritation potential as measured by hemolytic activity

**Figure 5:**
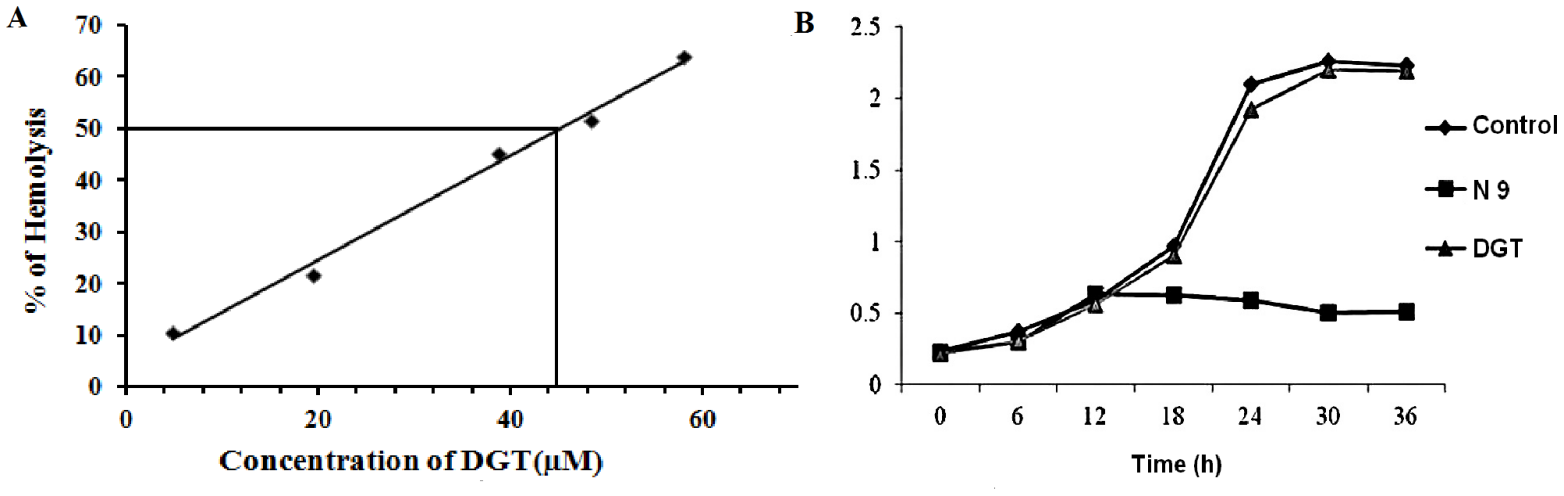
Irritation potential as measured by hemolytic activity and effect on *Lactobacillus acidophilus in vitro*. (A) Dose-dependent effect of DGT in rat RBCs. Isolated RBCs were incubated with varying drug dilutions, Triton X-100 and PBS. The extent of hemolysis was determined spectrophotometrically. (B) Effect of desgalactotigonin (DGT) and nonoxynol-9 on *Lactobacillus acidophilus*. [Optical density as the measure of turbidity denoting growth of bacterial colonies during 36 h of culture in the absence (control) and presence of DGT. There was a gradual increase in the growth of colonies that reached a plateau after 24 h of culture. Irrespective of the dose of DGT (19×MEC), the growth of bacterial colony was comparable with that of the control. N-9, however, exerted constant inhibition of bacterial growth throughout the entire culture period.]

The hemolytic activity of DGT for rat as depicted in (Fig. 5A) was determined by quantifying the hemoglobin released from the RBC following exposure to varying concentrations of DGT. At 47 µg/mL of DGT, 50% hemolysis occurred. That is one and half fold higher than MEC. RBC treated with 0.1% Triton X-100 served as positive control.

### Effect on *Lactobacillus acidophilus in vitro*

For this experiment, the control contains only lactobacillus strain with broth medium, DGT and nonoxynol-9 represents standard. This hour vs optical density graph (Fig. 5B) established that as compared with control, DGT up to 40 fold concentrations (for rat) and 19 fold concentrations (for human) of its MEC did not inhibit the growth of *Lactobacillus acidophilus* during the period of 36 h culture.

### Contraceptive efficacy in rats

**Figure 6:**
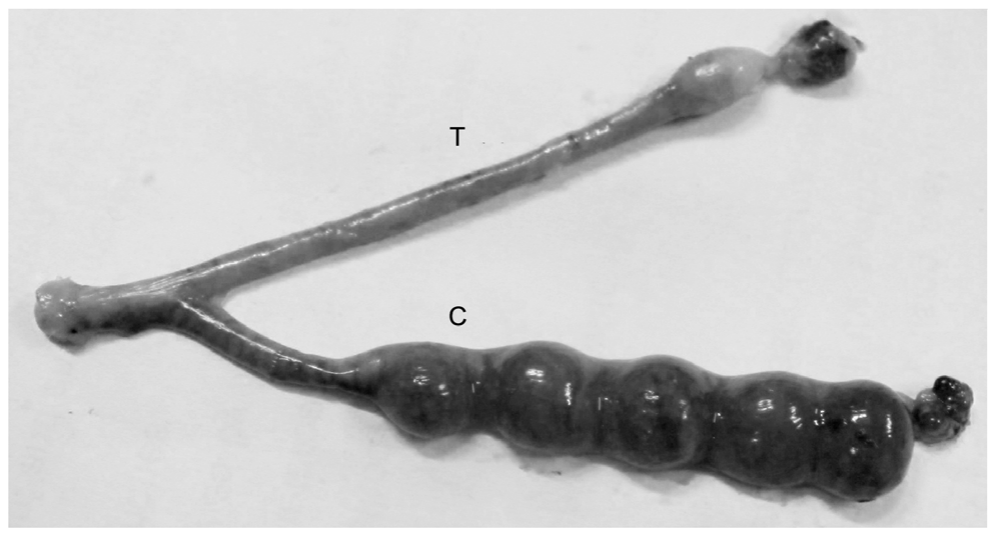
The contraceptive efficacy of DGT in Sprague-Dawley rats. Photograph of a uterus showing the presence or absence of implantation sites in the control [C] and DGT-treated [T] uterine horn of a Sprague–Dawley rat.

All the females mated successfully and no deviation of mating efficiency was observed. Not a single implantation site was observed in the DGT-treated horns on Day 10 of gestation. On the contrary, the number (mean±SEM) of implantation sites in the saline treated control horn showed the presence of 5±0.52 beads like embryonic swellings. On the parturation 4 normal pups were delivered by the other groups of rats. This observation (Fig. 6) suggests that even in the uterine milieu, DGT could effectively block sperm potential to reach and/or fertilize oocyte, while fertilization and implantation were unhindered in the control horn. The results suggest that DGT decreased fertility to zero.

### Ultrastructural study

#### Transmission electron microscope

**Figure 7:**
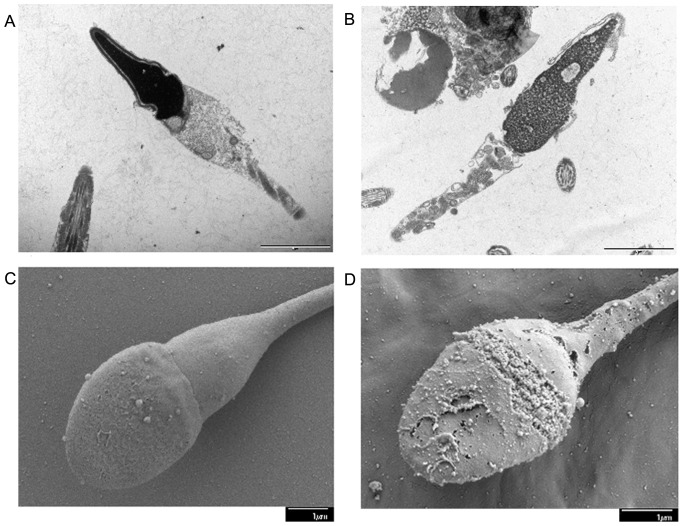
The microscopic ultrastructural changes in the sperm. Transmission electron micrographs of human sperm samples incubated in the absence or presence of DGT (at MEC). (A) Control spermatozoa show proper acrosomal cap with intact plasma membrane, while (B) DGT-treated spermatozoa exhibit dissolution of the acrosomal cap. High resolution scanning electron micrographs (×15000 and ×19000) of human sperm treated without and with DGT at MEC. (C) Control sperm shows intact acrosomal cap and plasma membrane around the head and neck regions, while (D) DGT-treated sperm demonstrates dissolution of the acrosomal cap.

Ultrastructural microphotograph showed considerable membrane damage in 100% of the DGT exposed human sperm. As compared with the intact plasma membrane surroundings the head of 80% of the control spermatozoa (Fig. 7A), all treated spermatozoa exhibited dissolution of the acrosomal cap, and expansion as well as separation of the plasma membrane from the nucleus (Fig. 7B).

#### Scanning electron microscope

Electron microscopic photographs of human spermatozoa are presented in Fig. 7C–D. As compared with intact plasma membrane and acrosomal vesicles of 92% of the untreated spermatozoa, all (100%) DGT treated spermatozoa exhibited disintegrated plasma membrane with damaged acrosomal cap of various degrees ranging from perforations and vesiculation to complete disintegration.

### Effect of lipid peroxidation on human spermatozoa

**Figure 8:**
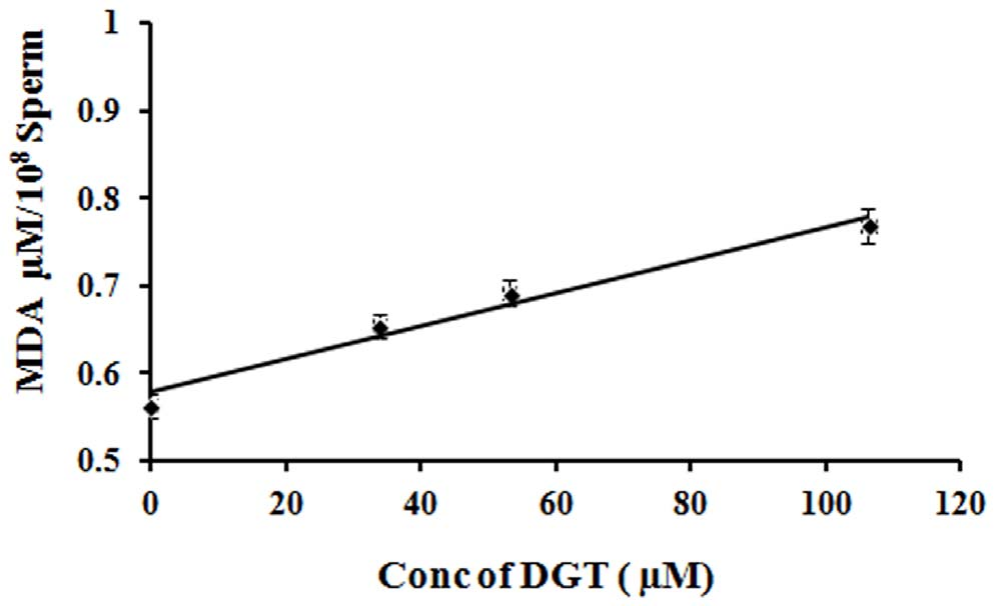
Effect of lipid peroxidation on human sperm. Generation of malondialdehyde (MDA) as a function of lipid peroxidation by human sperm treated with or without DGT at varying concentrations. Values on Y-axis represent mean ± SEM. values of five determinations (P<0.05). The graph shows dose-dependent increase in MDA generation following exposure of motile spermatozoa to DGT.

The spectrophotometric readings demonstrated a dose dependent increase in the concentration of malondialdehyde (MDA; µmol/10^8^ sperm) in concert with an increase in DGT concentration (Fig. 8). As analyzed by ANOVA, the MDA production by spermatozoa treated with DGT at MEC (0.697±0.0150 µmol/10^8^ sperm) was significantly higher (p<0.01) than that of control spermatozoa (0.563±0.0140 µmol/10^8^ sperm).

### MTT assay for cytotoxicity

DGT did not show any detectable cytotoxicity towards the human cervical cell line (HeLa) at MEC. The IC_50_ values for cytotoxicity of DGT and N-9 towards HeLa cells were 135.54 and 0.675 µg/ml respectively. The selectivity index of the compounds was calculated as a ratio of cytotoxicity IC_50_ to spermicidal EC_50_. DGT exhibited a selectivity index of 4.55, much higher than that of N-9. So, this cytotoxicity assay revealed a massive 529.06 level of safety for DGT compared with N-9 (Table 1).

**Table 1:** Spermicidal potential, cytotoxicity and selectivity index of DGT, N-9 [51].

Treatment	Spermicidal EC_100_ (µg/ml)	Spermicidal EC_50_ (µg/ml)	Cytotoxicity IC_50_ (µg/ml)	Selectivity Index (IC_50_/EC_50_)	Safety versus N-9
DGT	60	29.8	135.54	4.55	529.06
N-9	486.82	78.34	0.675	0.0086	1

### Induction of apoptosis by spermicides on human spermatozoa

**Figure 9:**
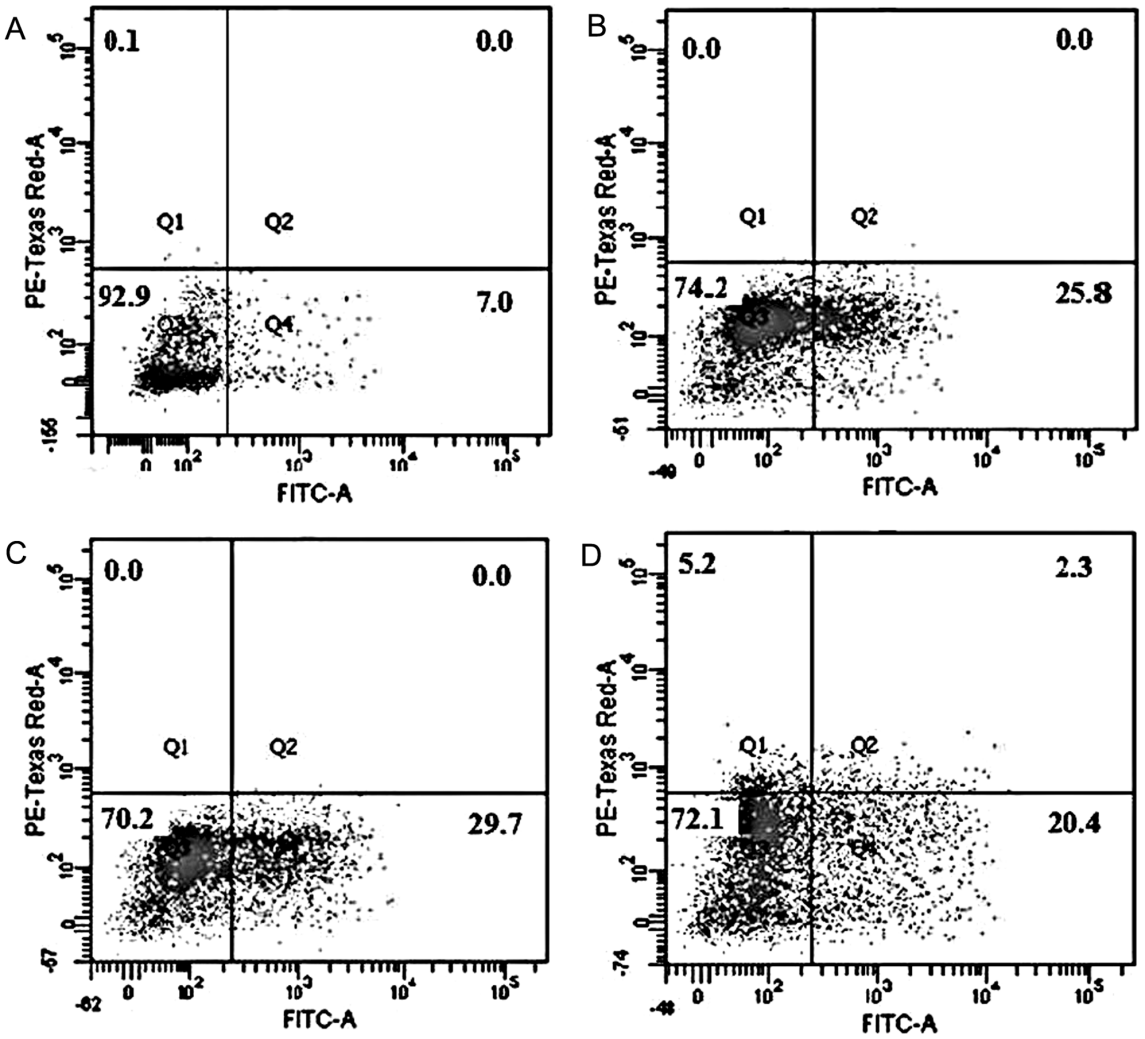
Apoptotic changes in plasma membrane. Representative images of apoptosis/necrosis induction by DGT in sperm cells, measured by Annexin-V/PI labelling and flow cytometry. Concentrations used were 29.01 µM, 58.03 µM and 106.38 µM of DGT against sperm for 3 h. [A. control, medium only, B. 29.01 µM DGT, C. 58.03 µM DGT, D. 106.38 µM DGT]. Cell population in four quadrants identified as: [lower left, live; lower right, apoptotic; upper right, necrotic; upper left, dead].

The selective spermicidal action of DGT (after 3 h incubation at different concentrations) is shown in Fig. 9 (Dot plot). By labeling with FITC-Annexin V (for detection of phosphatidyl serine on cell surface) and PI (for cell membrane integrity), cells were identified in four quadrants by flow cytometry: live (viable, LL), apoptotic (FITC stained, LR), necrotic (FITC+PI stained, UR), and dead (PI stained, UL). The data from the dual fluorescent labelling with Annexin V- FITC and PI showed that the control sample contained 92.9% viable, 7.0% apoptotic, 0.0% necrotic and 0.1% dead sperm (Fig. 9A). After treatment with DGT at EC_50_ (29.01 µM), the number of apoptotic cells rose significantly to 25.8%, with reduction in the populations of viable (74.2%) sperm cells (Fig. 9B). The figure increased to 29.7% after treatment with 58.03 µM DGT (Fig. 9C). The apoptotic, necrotic and dead cell populations increased to 20.4%, 2.3%, and 5.2% respectively after treatment with 106.38 µM DGT (Fig. 9D).

### Apoptotic changes in mitochondria on human spermatozoa

**Figure 10:**
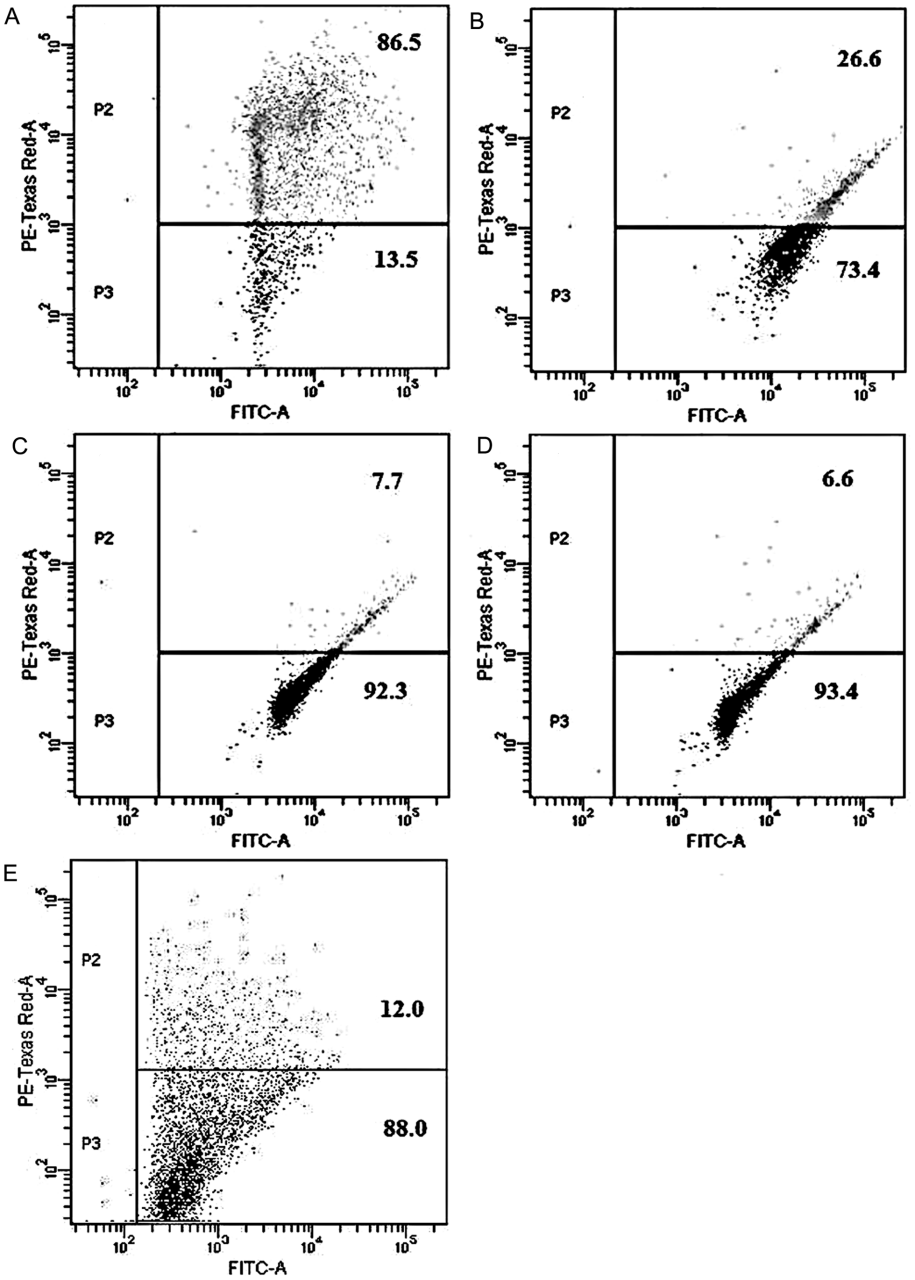
Apoptotic changes in mitochondria. Representative illustration of the depolarization of mitochondrial transmembrane potential of sperm cells by DGT, measured by lipophilic cationic dye JC-1 staining. Concentrations used were 29.01 µM, 58.03 µM, and 106.38 µM against sperm for 3 h. [A. control, medium only, B. 29.01 µM DGT, C. 58.03 µM DGT, D. 106.38 µM DGT, E. CCCP]. Cell population in quadrants identified as: [upper right, polarized; lower right, depolarized].

There was a significant decrease in mitochondrial transmembrane potential (ΔΨm) of sperm cells treated with the spermicide as indicated by an increase in the number of sperms exhibiting green fluorescence for JC-1. The decline was greater (79.9%) in case of DGT at 106.38 µM (Fig. 10D), but was 78.8% at 58.03 µM (Fig. 10C), 59.9% at 29.01 µM (Fig. 10B), and 74.5% for CCCP (Fig. 10E).

## Discussion

All the above experiments focus on the assessment of desgalactotigonin, which was isolated from seed extract of *Chenopodium album*, for its sperm immobilizing activity. Spermicidal activity, contraceptive efficacy and other related parameters of DGT as spermicidal agent were verified in a series of in vitro and in vivo experiments. Results revealed that the compound has sperm immobilizing property. At a concentration of 24.18 µM in case of rat and 58.03 µM in case of human, DGT caused 100% immobilization of sperm within 20 s with no revival of motility after subsequent 60 min of incubation in Baker's buffer at 37°C. So, the values of 24.18 µM for rat and 58.03 µM for human were considered as minimum effective concentrations (MEC) and used for further studies. DGT thus requires low concentration for the preparation of local vaginal contraceptive.

Plasma membrane integrity is directly dependent on membrane permeability, which is indispensable for effective sperm motility during transport and fertilization. HOS test is an indicator of the structural and functional integrity of sperm membrane [30], [31]. Plasma membrane is semi-permeable in living state; in hypo-osmotic condition, intact cell membrane facilitates free entrance of solvent in to the cell to attain osmotic equilibrium causing the cell to swell. Since the plasma membrane around the sperm tail fiber is more loosely bound as compared to the rest of the sperm, sperm cell is particularly susceptible to hypo-osmotic fluctuation and responds by curling. This characteristic feature was exhibited in >90% of the sperm in control set, while the DGT exposed sperm showed no such morphological distortion. This experiment illustrated that the functional integrity of sperm membrane was totally lost on exposure to DGT. This conclusion was additionally validated by using fluorescent staining method. Sperm viability was assessed by using a membrane permanent nucleic acid dye SYBR-14 (green, for live sperm) and PI (red, for dead sperm) [41]. In dual fluorescent staining method control spermatozoa appeared green while treated spermatozoa turned red. DGT treated sperm were almost 100% dead. All these observations reveal that DGT compromised sperm membrane integrity suggesting that there was an ultimate loss of functionality. Both TEM and SEM findings of human spermatozoa also illustrated that DGT treatment affected sperm membrane integrity with dissolution of outer acrosomal cap.

The above conclusion was supported biochemically by the observation of increased lipid peroxidation. Mammalian sperm cells present highly specific lipid composition comprising a high proportion of polyunsaturated fatty acids, plasmalogens and sphingomyelins. Malondialdehyde is a product of LPO that appears to be produced in relatively constant proportion to the breakdown of polyunsaturated fatty acids [38], [39]. Saponins have previously been reported to induce permeability change [42]. DGT being a saponin exerts similar mode of action. From this mechanism we deduced that DGT treated human spermatozoa totally lost their membrane integrity.

To evaluate the contraceptive efficacy of DGT we undertook in vivo investigation by using rat model. We wanted to utilize the advantage of bi-cornuate uterus with separate cervical opening of two uterine horns in rat. While one horn can serve as control, the other is the experimental one; both are exposed to identical systemic milieu. However, the rat model is not suitable for evaluating the efficacy of DGT as vaginal contraceptive because in this model mating involves disposition of sperm in the cervix, not in vagina [43]. Since sperm passes by the vagina to reach uterus, we introduced DGT directly into the uterine horn. The intrauterine applications of DGT in rats were significantly encouraging as fertility was reduced to zero on comparison with control.

Hemolytic index is a rapid screening assay of first order for the assessment of acute irritation potential of topically applicable microbicides or spermicides [44]. From this index, the subsequent doses which would be used for toxicity studies can be determined. At 45.5 µM of DGT, which is one and a half fold higher than MEC, 50% hemolysis occurred. However, further studies are required to explore the cytotoxicity of DGT.

The normal vaginal flora of healthy women is dominated by lactobacilli. Lactobacillus produces a number of compounds including lactic acid, hydrogen peroxide, lactacin and acidolin that maintain a low acidic pH (3.5–5.0) [45] and thereby protect against the pathogens that cause STIs including HIV [46], [47]. Currently used spermicides are mainly N-9 based. The vaginal spermicide nonoxynol-9 does not protect against sexually transmitted diseases and HIV in clinical situation but may in fact increase their incidence due to its non specific, surfactant actions [48]. But, in microbial studies, the effect of DGT on *Lactobacillus* culture was different. From the result it appears that a 20 fold higher concentration of its MEC has no significant inhibitory activity on the growth of *Lactobacillus acidophilus* during 36 h of culture period.

MTT assay was used to evaluate the cytotoxicity in human cervical (HeLa) cell lines. DGT has not only potent sperm immobilizing property but has precise, targeted action that kills 100% spermatozoa in 20 s without affecting human cervical cell line viability for up to 24 h in vitro culture. The IC_50_ concentration of DGT for cytotoxicity towards HeLa cells was much lower than that for N-9. This indicates that, in comparison with N-9, this spermicide specifically and selectively targets sperm cells, which provides a safety index of orders of magnitude in its favour. This is the most notable characteristic of this molecule.

Flow cytometry has been extensively employed for the analysis of acrosomal integrity, mitochondrial function, and motility of spermatozoa to determine the fertilizing potential of human sperm sample. It is an automated approach that can measure the amount of one or more fluorescent stains associated with cells in an unbiased manner, offering properties of precision, sensitivity, accuracy, rapidity, and multiparametric analysis on a statistically relevant number of cells. The finer aspects of spermicidal action on sperm cell were therefore studied at EC_50_ concentration by flow cytometry. The compound DGT induced apoptosis in sperm cells, which was characterized by an increase in FITC-Annexin V labelling of human spermatozoa. DGT treatment induces apoptosis without causing any necrosis (green and red fluorescence) of human sperm cells. This further indicates the mechanism based action of DGT on human spermatozoa that is different from general dissolution of cell membrane.

The mitochondrial transmembrane potential maintains the integrity of mitochondrial polarization for normal energy generation and dissipation, [49] and a significant drop in this potential indicates initiation of apoptotic process [50]. The depolarization of sperm mitochondria and induction of apoptosis by DGT once again reflects the difference in mechanisms of action.

Although this compound belongs to the saponin family, it has no detergent like property and the vaginal ecosystem remains unhampered. The uniqueness of this compound is that it has four sugar moieties and high molecular weight (1034), one and half times that of the synthetic molecule N-9 (MW 617), which indicates a lesser chance of absorption through vaginal epithelia. It has good solubility and MEC of this compound is comparatively low. It is possible that this rendered the compound more effective and bioavailable. Moreover, N-9 hampers the vaginal ecosystem by inhibiting the growth of lactobacilli, leading to an increased risk of developing sexually transmitted diseases and HIV. But DGT at twenty fold higher concentration than its MEC has no harmful effect on vaginal flora. In todays world most women prefer an antimicrobial spermicide. This compound can suitably replace N-9 in vaginal preparations to make them more acceptable for repeated use. The results of this study lead us to propose that this naturally occurring compound selectively kills sperm without hampering lactobacilli and HeLa cells at MEC, indicating its potential as a superior ingredient for a future contraceptive formulation. However, further studies related to pathogen killing, local toxicity and teratogenic properties are to be carried out to prove it as a prophylactic contraceptive agent.

### Statistical analysis

All biochemical observations were based on 5 independent experiments. Data were expressed as mean ± SEM or percentage. The results were analyzed by 1-way analysis of variance (ANOVA), and chi-square test, as applicable, using the Graph Pad Prism 3.0 software (Graph Pad software, Inc, San Diego, California). P<0.05 was considered significant.

## Supporting Information

Data S1 (DOC)
