# Supplementary material for: Spermicidal and Contraceptive Potential of Desgalactotigonin: A Prospective Alternative of Nonoxynol-9
Source: PLoS One. 2014 Sep 22;9(9):e107164. doi: 10.1371/journal.pone.0107164 (PMC4171379; doi:10.1371/journal.pone.0107164)
Supplement: Data S1 — (DOC) [file pone.0107164.s001.doc]

**Supplementary (S1) data**

The isolated minor products 3-*O*-β-D-Glucuronopyranosyl oleanolic acid, 0.0001% (**CA8**); 3-*O*-[β-D-Glucuronopyranosyl]-28- *O*-β-D-g-lucuronopyranosyl oleanolic acid, 0.0006%(**CA9**); and 3-*O-*[3′-*O*-(2′′-*O*-Glycolyl)-glyoxylyl β-D-glucuronopyranosyl] oleanolic acid, 0.0005% (**CA10**) were also evaluated for their spermicidal efficacy. The range of spermicidal MEC of **CA (8-10)** was found to be 75- 120µM, far less active compared to DGT. However, as the elucidation of its structure (3-*O*-β-D-Glucuronopyranosyl oleanolic acid) as well as revelation of the activity coincides with the literature (Das et al. 2011, Contraception) thus this data was not included in the manuscript. It is worthy of mention that the reported compound was from different plant source.

**Reference:**

Das N, Chandran P, Chakraborty S (2011) Potent spermicidal effect of oleanolic acid 3-beta-D-glucuronide, an active principle isolated from the plant Sesbania sesban Merrill. Contraception 83: 167-175.
